# 3D Digital Surveying and Modelling of Cave Geometry: Application to Paleolithic Rock Art

**DOI:** 10.3390/s90201108

**Published:** 2009-02-20

**Authors:** Diego González-Aguilera, Angel Muñoz-Nieto, Javier Gómez-Lahoz, Jesus Herrero-Pascual, Gabriel Gutierrez-Alonso

**Affiliations:** 1 Cartographic and Land Engineering Department. University of Salamanca, Hornos Caleros, 50, 05003 Avila, Spain; E-Mails: almuni@usal.es (A.M.-N.); fotod@usal.es (J.G.-L.); sabap@usal.es (J.H.-P.); 2 Geology Department. Faculty of Sciences. University of Salamanca, Plaza de los Caidos s/n, 37008 Salamanca, Spain; E-mail: gabi@usal.es (G. G-A.)

**Keywords:** Archaeological, remote sensing, caves, active sensor, laser scanning, point-based techniques, close-range photogrammetry

## Abstract

3D digital surveying and modelling of cave geometry represents a relevant approach for research, management and preservation of our cultural and geological legacy. In this paper, a multi-sensor approach based on a terrestrial laser scanner, a high-resolution digital camera and a total station is presented. Two emblematic caves of Paleolithic human occupation and situated in northern Spain, “Las Caldas” and “Peña de Candamo”, have been chosen to put in practise this approach. As a result, an integral and multi-scalable 3D model is generated which may allow other scientists, pre-historians, geologists…, to work on two different levels, integrating different Paleolithic Art datasets: (1) a basic level based on the accurate and metric support provided by the laser scanner; and (2) a advanced level using the range and image-based modelling.

## 1. Introduction

Accurate surveying of cave passages, chambers and the shape of their walls has always been a priority issue before attempting to describe or understand processes takes place underground, whether they are related to the origin and formation of the caves, or to the human activities that may have occurred in them. The new computerized digital techniques provide tools that increase notably the efficiency and the accuracy of the surveys and provide the basic grounds for any further research. In this work we present the integrated use of non invasive techniques to document two of the most significant Paleolithic caves in northern Spain, especially focused on prehistoric wall paintings and engravings.

The Spanish Cantabrian Mountains together with Southwestern France and the Pyrenees concentrate around 95% of all known Paleolithic settlements in Europe. The uncontrolled increase of visitors, as well as the natural damage caused by time, has negatively affected these extremely delicate art forms. Classic cave surveying has been always performed through compass and tape techniques, and in very rare cases precise surveying instruments have been used in the survey of large portion of caves. In addition, the aforementioned techniques do not provide an accurate 3D representation of the underground passages and chambers. Therefore, it is essential to integrate the new available technologies and methodologies in order to allow its preservation and to provide scientists with an accurate, non invasive, surveying tool that efficiently enables the 3D reconstruction of this Cultural Heritage wealth.

The studied caves are two of the seventeen decorated caves of the Paleolithic age that were inscribed in 2008 into the World Heritage property list, as an extension to the Altamira Cave, inscribed in 1985. The property will now appear on the World Heritage as Cave of Altamira and Paleolithic Cave Art of Northern Spain. The property represents the apogee of Paleolithic cave art that developed across Europe, from the Urals to the Iberian Peninsula, from 35,000 to 11,000 BC.

### 1.1. Archaeologists' Needs and Requirements

The different analog techniques for documenting engravings and pictographs that are discussed in the following subsection have provided results of variable quality but sharing two common features: a bidimensional character and the lack of spatial content. Photographs, sketches, drawings and artistic representations have the limitation of being bidimensional documents. However, it is well-known that the Paleolithic Art is not a “flat art”. In fact, our forefathers made good use of the free-form shapes of the rocks with the aim of fitting the drawings of abstract forms and animal's figures, achieving more realism and maybe meanings that have been not yet correctly decoded.

In the last 10 years geomatic techniques have been offering a relevant contribution for archeologists' research. The possibility of obtaining rigorous metric information on cupped archaeological structures in a non-destructive way constitutes a precious advantage for planning and optimizing excavations in already known sites. Particularly, four main advantages could be outlined:

- First, the acquired information can be integrated with digital images taken from terrestrial, aerial or satellite platforms, in order to effectively support the investigation and the location of those archaeological sites which are partially known and of those which have been not yet discovered.
- Second, archaeological findings can be represented and geo-referenced in a spatial context according to their dimensions (for example, an open gallery, a chamber with elevated walls, a complex panel with convex and concave shapes, etc).
- Third, the three-dimensional character of the acquired dataset provides new elements of artistic interpretation through the three-dimensional analysis of the archaeologist.
- Fourth, digital documentation allows for a more effective and wider dissemination of the results obtained in different archaeological campaigns, thanks to the improvements in representation and visualisation capabilities related to the generation of photorealistic models and virtual animations.

The case study presented in this paper constitutes a meaningful example of the effectiveness of the geomatic and non-destructive methods in meeting archaeologists' requirements. For example, digital image processing is used for visualization enhancement and for automatic extraction of features making easier the interpretation and identification of archaeological characteristics. Moreover, the developed geomatic approach has proved to be an effective and useful tool for archaeologists who want to perform their research studies. The often difficult environmental conditions and the presence of a complex geometry (free-form shapes) affect negatively the survey. Nevertheless, using proper field procedures to collect data and good processing techniques it is possible to obtain the maximum content of information.

### 1.2. From Classical Techniques to New Technologies: Previous Work

Recording the fine details of rock art using classical techniques such as drawing, tracing, rubbing or photography suffer from several drawbacks. Free hand drawing over the surface is a simple, easy and low-cost technique providing only a two dimensional sketch which is generally inaccurate. Once the visibility of the petroglyphs is assessed the next step is to trace the figures. The easiest and best way to record petroglyphs is to use transparent plastic field sheets, commonly cut to standard sizes. Though usually adopted in the Archaeological field, this method creates large volumes of media which have to be photographically reduced for more efficient storage and manually assembled to obtain the complete surface. Placing a grid over the object and transferring detail one square at a time solves for the physical reduction problem directly, however it is an invasive procedure, requiring the physical touching of the art which entails patience and extensive field time [1].

Other recording techniques, such as rubbing (or frottage) are not as precise as tracing: the superimposition within the figures and the distinction between the carvings and the natural fractures are often not clear. In any case the frottage, if repeated continuously on the same figures, can be considered a destructive technique and can cause the abrasion of the pecking. Massive utilization of these methods is justified because of low cost of traditional recording.

More recently, geomatic documentation techniques have emerged. Such techniques are indispensable tools for the conservation and preservation of rock art. The methods and equipment commonly used for the documentation and surveying of rock art are: topographic, photogrammetric and laser scanning.

- *Topographic methods*. Topographic work in subterranean sites [2] has been limited until lately. Methods based on angles, distances and height variations measurements are really useful in the caves and karst studies when possible. The equipment used is composed of accurate and appropriate theodolites or total stations. When the point to be determined is inaccessible indirect methods such as single or multiple intersections are used [3]. New developments have been incorporated into total stations, such as the measurement of distances without reflector element by means of laser rays, reducing the fieldwork considerably. However, the morphologic complexity of caves not only affects the data acquisition, but also the way these datasets are represented, usually with simple cross-sections and ground plans. As a result, the documentation of caves was usually performed with expeditious methods such as: compass, clinometers, tap measure and supported by the use of a specific symbology.
- *Terrestrial photogrammetric methods*. By correcting images from lens and perspective distortions, photogrammetry allows to extract accurate measurements from photographs. A scaled drawing or 3D model can then be made on the computer directly from the corrected images. In this context, photogrammetrists have developed several approaches to record rock art in 3D: from the classical techniques based on stereoscopic vision to multiple image-based modelling supported by bundle adjustment. Nevertheless, although the acquisition of digital images remains a universal and low-cost alternative, particularly for simple recording and qualitative use, the extraction of quantitative data from photographs is less common in the rock art framework where the fieldwork conditions are very specific and the geometry presents great complexity, requiring time, skill and knowledge related to photogrammetry. Trying to improve this last drawback, [4] present a low-cost methodology that enables to generate both accurate and dense DEMs and orthophotographs by non-expert in photogrammetry. These data are able to record detailed morphology, generate three dimensional visualizations and the ubiquitous fly through model. The methodology was developed and tested using a series of case studies, representing a diverse selection of aboriginal rock art. On the other hand, in a series of related projects [5] and [6], Ogleby and Rivett demonstrated the benefits of photogrammetry for recording rock art, particularly pictographs. Fieldwork was conducted at a series of sites around Australia and their “*Handbook of Photogrammetry*” was a key text of its day describing how to perform a photogrammetric survey in archaeological field. Afterwards, [7] has continued to demonstrate the benefits of photogrammetry to a wider archaeological audience including the Ayutthaya temple in Thailand and the Olympiad. In these two examples, an important final product has been the virtual model, enabling the visualization of the site from any perspective.
- *Laser scanning methods*. In the last years, the emergence of the Terrestrial Laser Scanner (TLS) as an effective surveying technology has opened up new perspectives for the recording and 3D reconstruction of rock art, as well as a multidisciplinary framework in which professionals and researchers with a different background have the opportunity to take part. Based on laser technology, TLS systems allow to perform dense and accurate measurements of real objects in very short time, producing the well known “pointcloud”. Two different principles for distance measurement are in use: ranging lasers using the “time-of-flight” or “phase measurement” principle, and instruments using CCD cameras where distance measurement is based on the principle of “triangulation”. The time-of-flight scanners base their ranging measurement determining the time-of-flight of a light pulse, i.e., by measuring the travelling time between the emitted and received pulse. The phase measurement scanners base their ranging principle measuring the phase difference between the transmitted and the received signal backscattered from the object surface. This method is applied with lasers that continuously emit light. Ranging scanners are able to measure much longer distances than instruments that work by triangulation. They are, however, less accurate and especially so at close range. The accuracy is between one millimetre and two or three centimetres, depending to some extent on the distance between the object and the scanner. This ranging accuracy is a key parameter. According to time-o-flight scanners, ranging accuracy basically depends on the time interval measurement, while for the phase measurement scanners this accuracy fundamentally depends on the wavelength of the ranging signal. Triangulation scanners are based on a simple triangulation principle. A light spot or stripe is projected onto an object surface and the position of the spot on the object is recorded by one or more CCD cameras. The angle of the laser beam leaving the scanner is internally recorded and the fixed base length between laser source and camera is known from calibration. The distance from the object to the instrument is geometrically determined from the recorded angle and base length. This type of scanner reaches 3D point standard deviations of less than one millimetre at very close range (less than 2 meters). The ranging accuracy depends on both the length of the scanner base and the object distance. With a fixed base length, the standard deviation of the distance measurement will increase in proportion to the square of the distance.
  One way or another, laser technology has being widely used so far in several scientific fields such as architectural heritage, civil engineering, industrial reconstruction or geology [8-11], but its use underground is not very common. Caves and karst studies represent a specific case (given the geometric and environmental conditions) with great social relevance since cave preservation seems more vulnerable to subsequent events, agents and processes. Nevertheless, this last aspect has contributed positively to local authorities starting to be more concerned about the special importance of recording and modelling rock art using non-destructive techniques, such as laser scanning. In this sense, in [12] use a triangulation-based laser range scanner to physically replicate the cave at Altamira in Northern Spain. Numerous modeling and CAD software tools were used and the data was fed into a milling machine to create the cave replica. The rock art was physically painted onto the replica. In [13] a case of study for recording and monitoring the rock art erosion based on triangulation laser scanner is presented. The 3D laser scanner method is able to produce a precise digital model of the carved rock and provides accurate geo-referenced data in sub-millimetre resolution, thus allowing the quantitative assessment of surface erosion and deterioration of the rock art by comparison with future recordings. In [14] the authors present data processing techniques for the recording of rock art using a TLS. Particularly, they use a triangulation laser scanner for the documentation of petroglyphs. The most interesting contribution is their comparison with traditional rock art recording methods (rubbing and tracing). However, a point that is not investigated in this paper is the possibility of establishing an integration with terrestrial photogrammetry in order to process and visualize the results. [15] uses a phase measurement laser scanner and modelling software to produce an accurate three-dimensional model of a carved rock surface from the Upper Palaeolithic site of Cap Blanc in southwest France. However, the approach presented is really simple since only the geometry provided by the laser is processed. Additional information such as texture is not considered so far and the methodology presented only applies the classical laser scanning workflow. More recently, [16] establish a comparison between classical photogrammetry (stereoscopic) and modern laser scanning. Particularly, separate surveys are carried out using time-of-flight and triangulation laser scanners, as well as using stereo-paired photography. The resulting mesh surfaces are analyzed and compared. The strengths and weaknesses of each approach are scrutinised, alongside a practical and methodological evaluation of each technique in view of the aims of the survey. Finally, in a more sophisticated approach, in [17] an original method based on Multi-Resolution Digital 3D Imaging System is presented and put in practise into the Grotta dei Cervi (Italy). They used a prototype multi-resolution 3D laser imaging scanner that combines high-accuracy 3D laser imaging, very high-resolution perspective colour projection, and on-site geometric calibration of the intrinsic and extrinsic parameters. However, this approach needs a camera attached to a laser scanner in order to obtain an automatic colour texture projection.

In this paper we have attempted a multi-sensor approach, which by using the appropriate sensors and algorithms to acquire and process the data, will generate an accurate complete three-dimensional representation of that site easily and rapidly. The paper presents the following structure and organization: In Section 2 the full methodology using different sensors is explained in detail, especially the data processing step which applies a co-registration of all datasets. Section 3 shows some experimental results tested and applied to both Paleolithic caves. A final Section is devoted to outline some conclusions and future works.

## 2. Methodology

TLS technology is the most accurate up to date approach for surveying and 3D reconstruction of complex surfaces such as cave passages and chambers, providing a wealth of high quality results in a very short time. Nevertheless, TLS technology has also some drawbacks: (1) the high-cost of the required hardware; (2) the complex processing of the dense datasets and (3) its poor colour resolution. First two issues are easily solved by hiring or using shared equipment and using enough computing power while the latter would require the use of a high-resolution digital photographic camera in order to complement the laser model with colour, geometry and texture information.

In order to precisely model large complex natural surfaces three main issues need to be accomplished: (1) data recording from various types of sensors (laser scanner, digital camera and total station); (2) registration of all data in a common reference system; and (3) 3D reconstruction of the complex scene using hybrid approaches.

**Figure 1.**
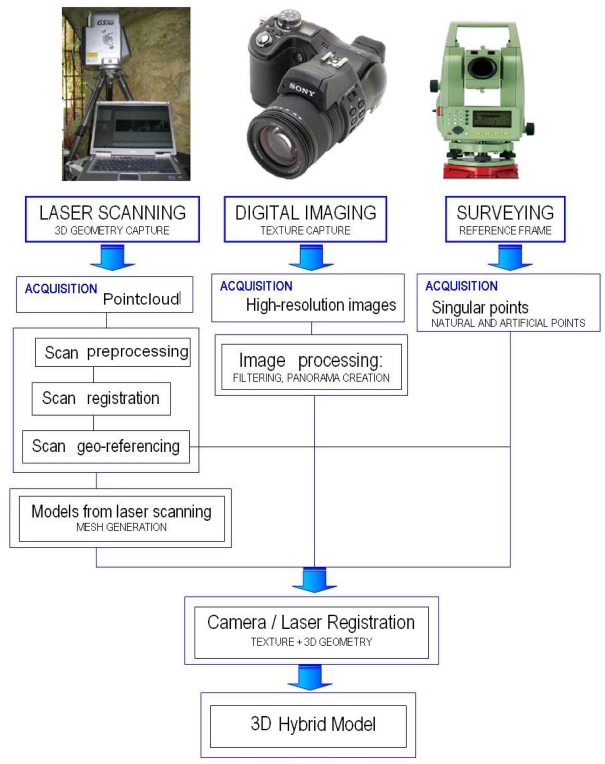
Multi-sensor approach applied at caves.

Figure 1 summarizes the full methodology developed for data acquisition, registration and modelling.

The methodology developed to acquire complete datasets for precise representation of complex surfaces is divided into two stages: field work and data processing. While the former deals with the equipment setup and data collection, the latter is focused just on data processing. During this stage, the information is processed in order to deliver plans and 3D models reproducing the original structure on a certain scale and the different outputs are assessed and evaluated.

### 2.1. Equipment Employed

In order to accomplish the data acquisition as well as the data processing we have used the following equipment:

#### Measuring sensors

- A Trimble GS200 TLS based on the time-of-flight principle. This instrument is motorized and allows for angular and distance accuracies of about 2.5 mgon (0.00225°) and 1.5 mm at distances below 50 m, respectively. The scanner uses an auto focus method for the laser which shows to be very useful mainly for close range applications. This feature guarantees a constant small laser spot even at different distances within a scan. The beam diameter varies from 0.3 mm at 5 m up to 1.5 mm at a distance of 25 m. In addition, special head gear, Manfrotto 400, is adapted to the laser scanner structure in order to overcome vertical range limitation.
- A Leica TPS 800 laser total station. This instrument is used in order to provide a common reference system according to the local reference frame established with each sensor independently. The total station has the following technical features: magnification 30×; electronic level sensitivity: 20 in/2 mm; twofold axis compensator; measurement accuracy of distances with prism: 2 mm + 2 ppm; measurements accuracy of distances without prism: 3 mm + 2 ppm.
- A non metric digital camera, Sony DSC F828. This camera is fitted with fixed-focus optics granting stability in the inner orientation. The CCD image sensor provides image sizes of 3,360 × 2,460 pixels, storing files in JPEG format.

#### Specific software tools

- An educational laser scanner software [18], which allows a co-registration and hybrid modelling of dataset provided by laser scanners and conventional digital cameras.

### 2.2. Field Work

Field work can be summed up in the following stages:

- Planning: before initiating any documentation work, objectives must be defined in a clear and accurate way. Poorly defined objectives will clearly affect the quality of the results, especially when recording subterranean places where the different conditions that can be found under ground could be really adverse: from physical features i.e. a difficult access, reduced illumination and high humidity, to technical features that have to be solved with an adequate methodology and specific instruments.
- Camera calibration. Considering that perform a calibration of the camera directly (self-calibration) from project images at caves or subterranean places is often not possible, a precise and reliable camera calibration including all lens distortion parameters will be required before data capture. With this aim, a specific camera calibration is performed on a laboratory using a grid of surveyed control points. Precise camera calibration including all lens distortion parameters is essential for sensor integration, especially if we try to guarantee more automatism and quality in the process.
- Range data acquisition: TLS dataset are taken following the basic principles defined in [19]. As a result, two types of range data are acquired: *global range data* set up with an average grid resolution of 20 mm at 10 meters of distance, with the aim of recording the whole cave geometry; *detailed range data* set up with an average grid resolution of 2mm at a distance of 10 meters, with the aim of recording the Paleolithic engravings and paintings with enough high accuracy.
- Image data acquisition: photographs are taken according to the basic principles of photography. The following aspects should be taken into account: using the right lighting conditions, avoiding shadows, reflections, backlights or burned photographs; calculating the depth of field, the speed of the camera and framing, which can vary depending on the type of images. As a result, three types of images are acquired: *multiple and convergent images* are taken following specific rules [20] and provide the input data to perform a photogrammetric image-based modelling; *independent high-resolution images* are used to generate metric texture maps from detailed range data; *panoramic images* are used to provide a 360-degree panorama background at the caves and to map the textures over the global range dataset. These panoramic images are acquired incorporating a specific lens (fish-eye) to the digital camera together with a specific pano-head (MrotatorTCPShort) and its tripod.
- Control points acquisition: a set of artificial targets (planar and spheres) are surveyed with total station with a twofold purpose: to register datasets in a common reference system and to geo-reference datasets into the archaeological frame provided by the prehistorian. Besides, this reference system provides a definition of the vertical direction without ambiguity.

Using this multi-sensor approach, the field work required a total time of eight hours. As a result, a full dataset supported by 3D and 2D information was arranged, ready to be processed.

### 2.3. Data Processing

Process data coming from different sensors is increasingly being used for complex sites modelling. In fact, there is a growing body of work on the integration of laser scanners and digital cameras. In these integrated systems, the laser scanner usually represents the dominant device, while the digital camera is primarily used as data source for the texturing of surfaces or pointclouds. Beyond this, the use of images for the automatic registration of laser scanner datasets has been suggested in previous approaches. Particularly, [21] develop a new approach for mapping and blending textures on 3D geometries. The system starts from a 3D mesh which represents a real object and texture detail acquired via a common photographic process. Both datasets are integrated based on initial rough registration. However, this approach requires manual interaction and is applied to small objects. [22] presents a novel stereo-based method for registering colour and range images acquired from externally uncalibrated sensors. The multi-sensor alignment problem is solved by processing invariant features such as corner, edges or contours which are extracted from the raw data (range and intensity values). The benefit of a feature-based approach is that it abstracts the data and thus simplifies the search for the registration parameters. The authors of [23], in their Great Buddha work, use reflectance edges obtained from the 3D points and match them against edges in the image to obtain the camera position. They align edges extracted from reflectance images with those in colour images so that the 3D position error of those edges is minimized by iterative calculation. Nevertheless, this approach has been focused on small and simple objects. More recently, [24] exploit the power of image modelling based on a single image to obtain an automatic co-registration of laser scanner and uncalibrated digital camera. Particularly, the problem of image registration is solved automatically through 2D and 3D point correspondences which are matched based on a search of spatial invariants: two distances and one angle. However, several input considerations such as especial targets, vanishing points and geometric constraints have to be taken into account. In [25] a strategy using a coded target placed on the object, which are registered by a calibrated digital camera, rigidly attached to the laser scanner was developed. An automatic process is applied to solve the spatial position and orientation of the camera within the laser scanner coordinate system. The identified coded targets are used to apply a 3D similarity transformation. However, this approach needs a camera attached to laser scanner and some code target placed on the object.

The data processing method presented in this paper (Figure 1) represents an original production workflow applied to Paleolithic rock art. Particularly, data processing is carried out through an automatic co-registration process which integrates images and point clouds. The co-registration approach developed tries to deal with two different images: a high-resolution image acquired with a conventional digital camera and a range image provided by TLS. To this end, a two-steps hierarchical co-registration strategy is developed as follows:

In the first step feature extraction and matching process based on interest points are applied to both images (high-resolution and range). The main goal is the semi-automatic extraction and matching of features (interest points) between high-resolution and range image. In particular, a Feature Based Matching (FBM) is applied as follows:

- A *feature extraction process* based on interest point detection, particularly Förstner operator [26] is used. This operator exhibits the following advantages compared to other alternatives: high accuracy and reliability in the localization of the interest points. Besides, different thresholds and parameters are established such as the ellipse circularity parameter and the point weight as expressed by the following formulae (1), so a non- maximum suppression is applied in order to extract the interest points
  (1) $$q=1-{\left(\frac{\lambda_{1}-\lambda_{2}}{\lambda_{1}+\lambda_{2}}\right)}^{2}=\frac{4\cdot \left|N\right|}{tr^{2}(N)}\;w=\frac{\left|N\right|}{\frac{1}{2}\cdot tr(N)}$$
  where *q* is the ellipse circularity parameter, *λ_1_* and *λ_2_* are the eigenvalues, *w* the point weight and **N** the Hessian matrix. The use of *q*-parameter allows us to avoid features which are not suitable for the purposes of the co-registration.
- A *robust matching* strategy that combines various scientific proven techniques (ABM-Area Based Matching and LSM-Least Square Matching) is applied.
  Firstly, for each extracted point in the high-resolution image an area-based matching is computed using the cross-correlation coefficient (2). In ABM, each point to be matched is the centre of a small window of pixels (patch) in a reference image (template) which is statistically compared with equal sized windows of pixels in another (target) image. Particularly, area-based image matching is performed with cross-correlation as a similarity measure (2).
  (2) $$\rho =\frac{\sigma_{HR}}{\sigma_{H}\sigma_{R}}$$
  where *p* is the cross-correlation coefficient, *σ_HR_* is the covariance between the windows of high-resolution and range image; *σ_H_* is the high-resolution image deviation and *σ_R_* is range image deviation. Interest points matching are based on proximity and similarity of their intensity neighbourhood.
  Cross-correlation works fast and well if the patches contain enough signal without too much high-frequency content (noise) and if geometrical and radiometric distortions are minimal. To overcome these problems, the approximations found with cross-correlation are refined considerably using Least Squares Matching (LSM) [27], which provides precise and sub-pixel location of the matching elements, considering image reshaping parameters and radiometric corrections. The location and shape of the matched window is estimated with respect to some initial values and computed until the grey-level differences between the deformed patch and the template one reach a minimum (3),
  (3) $$v=F(\bar{x},\bar{y})-G({\mathrm{ax}}_{0}+{\mathrm{by}}_{0}+\Delta x+{\mathrm{cx}}_{0}+{\mathrm{dy}}_{0}+\Delta y)r_{1}+r_{0}\to \min$$
  where *F* and *G* represent the reference and corresponding searching images respectively, *a,b,c,d,Δx* and *Δy* constitute the geometric corrections of an affine transformation, while *r_1_* and *r_0_* are the radiometric corrections, more specifically contrast and bright corrections respectively.

In the present case, images matching is always performed considering pairs of images (range and high-resolution) in order to retrieve relative camera parameters. Due to the different intrinsic characteristics of both images, the results present several mismatches which could degrade the final solution. Therefore, in some circumstances the user interaction is required in order to obtain precise and reliable results. Particularly, in the case of “Peña de Candamo” cave, a high-resolution image (3,264 × 2,448 pixels) and a range image (generated with 4 millions of points approximately) are processed. A total of 1,004 interest points are extracted, while only 804 are matched. In the case of “Las Caldas” cave, a total of 1,137 interest points are extracted of whom 983 are matched. Likewise, in both cases the matching process is applied with the following input parameters: cross-correlation factor (0.7) and size of search kernel (15 pixels).

Afterwards, in the second step, a co-registration of pair of images (range and high resolution) is computed following a two-step approach:

- An *estimation step* which allows us to obtain a first approximation of co-registration parameters based on Direct Linear Transformation (DLT) [28]. This transformation is linear with respect to the transformation parameters and is constituted by eleven parameters (three for the interior orientation, six for the exterior orientation and two additional parameters that contains a scale difference between the two images camera axes and non-orthogonality of these axes). Hence, the DLT is most suitable for the processing of non-metric images, such as amateur cameras. Since, it is not, however, a least squares adjustment its accuracy is limited and thus the results of DLT are used as approximations for a subsequent adjustment based on central perspective relations.
- A *computation step*, in which a re-weighted least square adjustment is applied. A re-projection strategy based on collinearity condition allows us to refine the DLT solution and thus to compute accurate and reliable co-registration parameters.

This step focuses on how to use the matching results to compute the camera resection parameters in relation to the laser scanner. The camera resection parameters are determined by the camera rotation angles (*ω, φ, κ*) around *X, Y* and *Z*, respectively and the camera pose (*X_0_, Y_0_, Z_0_*). In the present case, this process is performed for each image individually. In other words, once a common reference system is obtained for range dataset, each high-resolution image is co-registered individually through the approach described above. The resulting parameters are used for computing the texture-maps for each model. The final average standard deviation of the camera position *σ_xo,yo,zo_*, which measures the goodness-of-fit in the camera resection, is 0.03 m for the first cave and 0.02 m for the second cave, which is relatively good having in mind the circumstances of both cases, together with the own laser scanner performance [29,30]. In this sense, in [29] authors try to calibrate the laser scanner, providing some remarkable facts: the distances are biased by an additive constant and this constant depends on the colour of the surface. In a similar context, in [30] authors perform an accuracy and reliability control of laser scanning workflow based on high-precision topography. Particularly, based on a free network adjustment of control points represented by artificial targets, a dimensional analysis using distances is computed. Also, in this case interesting facts are outlined, mainly the presence of additional errors in the laser scanning workflow. More recently, with the aim of minimizing these unexpected errors, in [31] the author proposes a calibration model which incorporates an estimation of these additional errors.

Taking into account the remarks made before, these additional errors could have affected to the registration results, as well as to image observations. In fact, the average standard deviation of unit weight of image observations, *σ_0,xy_*, estimated from the spatial resection, is 1.3 pixels for the first cave and 1.5 pixels for the second cave. In addition, image observations are affected by the own matching process which is performed between images completely different (range and high-resolution images).

Last but not least, a ground truth has been added based on topographic techniques and the use of special targets for checking the registration parameters. That is to say, spatial invariants (distances and angles) between combinations of targets have been obtained once the resection camera parameters are computed. This dimensional analysis has been compared with that one provided by topography, obtaining good results.

Finally, once both dataset have been registered, we must geo-reference them to the ground coordinate system defined by the prehistorians. To this end, a set of control points (special targets) surveyed with the total station and acquired with the laser scanner and the digital camera are manually identified. A spatial 3D Helmert transformation is based on the spatial rototranslation between two different coordinate systems, as shown in equation (4)

(4) $$\left[\begin{array}{c} X\\ Y\\ Z \end{array}\right]=k\cdot R\cdot \left[\begin{array}{c} x\\ y\\ z \end{array}\right]+\left[\begin{array}{c} X_{0}\\ Y_{0}\\ Z_{0} \end{array}\right]$$

where (*X, Y, Z*) are the coordinates in the ground system, (*x, y, z*) are the coordinates in the local system, *k* is the scale factor, *R* is the rotation matrix and (*X_0_, Y_0_, Z_0_*) the translation vector.

In this project, an intense processing season was performed by six researchers and technicians in order to obtain final results. A total of three months were required for the data acquisition and modelling of all 3D and 2D information coming from both caves.

## 3. Application to Archaeology Study Case: “Las Caldas” and “Peña de Candamo” Caves

**Figure 2.**
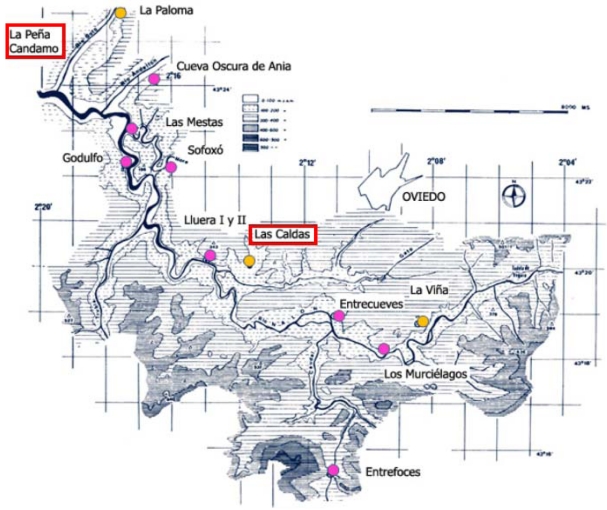
Location map: Nalon river middle valley. Main Paleolithic archaeological sites and rock-art stations.

The above explained methodology is applied to the Paleolithic caves “Las Caldas” and “Peña de Candamo”. Both caves are situated in the north of Spain, in a region known as Asturias (Figure 2) developed in Carboniferous massive limestones of the Caliza de Montaña formation.

The first cave, “Las Caldas”, is situated on the right side of Nalon Valley, in a small village known as Priorio, six kilometers away from Oviedo. The main entry is oriented towards the south-west, in the lower part of a valley transited by “Las Caldas” river. The surface of the cave is around 25 m^2^ composing of a main corridor with two different enlargements. As a Paleolithic settlement, the relevance of this cave is focused mainly on the collections that have been discovered there which belong to the most important ones in relation to Mobiliar Art in Europe, dated at the Middle Magdalenian period. These collections gather quality and unique pieces such as petroglyphs or pictographs engraved over stones, bones or even ivory. The parietal engravings, located at the cave lobby, appear sliced due to the slip of lateral walls, which occurred during the sedimentation of the levels belonging to Middle Solutrean. This circumstance – the possibility of dating, through stratigraphy, the petroglyphs- constitutes a not very common case in the Paleolithic Parietal Art.

The second cave, “Peña de Candamo”, is situated at the top of a lime hill dominating the Nalon Valley, with a main entry oriented towards the north-west. The cave presents three different halls or levels: “La Gran Sala”, a great hall situated 60 meters from the main entry; “El Camarín”, a hall situated 20 meters above the great hall; “La Sala Baja de los signos rojos”, a small hall situated near the main entry which preserves linear strokes and punctual marks with a difficult dating. More in detail, the engravings found in “La Gran Sala” - pictographs and petroglyphs in red and black colors - are located on a big panel at the right side of the great hall, known as “Muro de los Grabados”, and on two smaller panels, one on the left wall, with a pictograph of a goat, and the other one on the right side with pictograph of a horse in red. A last relevant aspect on “El Muro de los Grabados” is related to the presence of engravings depicting three sea mammals. This is particularly important since several seal teeth were found at “Las Caldas” cave. These datasets reinforce the parallelism between both caves and make evident the existence of movements towards the seacoast (40 or 50 km away), as well as the hunting of seals in the Nalon Valley itself.

With relation to the results obtained, two levels of information are generated: (i) a basic level based on the metric support provided by the TLS, which allows the archaeologists to obtain any metric measurement easily. Particularly, a spatial surface resolution of 2-3 mm is reached for the detailed laser scanner models, while a spatial surface resolution of 1-2 cm is achieved for the global models; (ii) and advanced level based on the co-registration of all datasets under a common reference system, which allows the prehistorian to get an integral and photorealistic model ready to be interpreted and analyzed.

**Figure 3.**
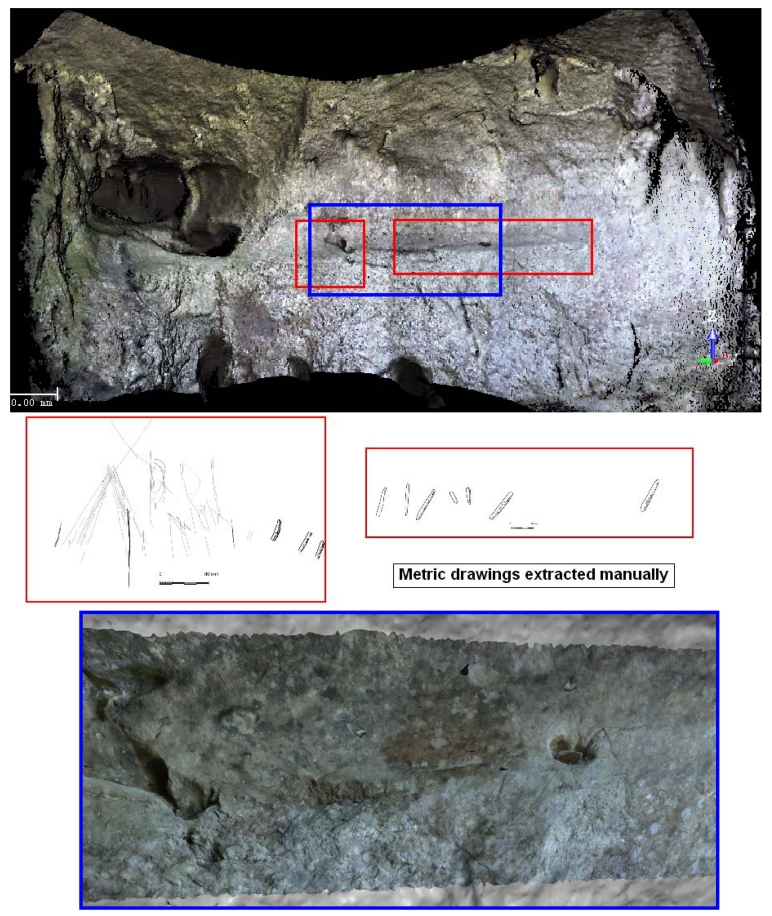
Detailed 3D hybrid model together with vector scale drawings (red rectangles) extracted from this model. Close-up of the 3D model (blue rectangle).

The above figures illustrate some of the results obtained. High-resolution textured and metric models based on the developed co-registration approach are generated. Figure 3 illustrates the results achieved after the application of our method to an emblematic panel at “Las Caldas” cave. In particular, image-based modelling has been applied to incorporate geometric constraints (breaklines) to the laser model, as well as to reconstruct several emblematic engravings.

**Figure 4.**
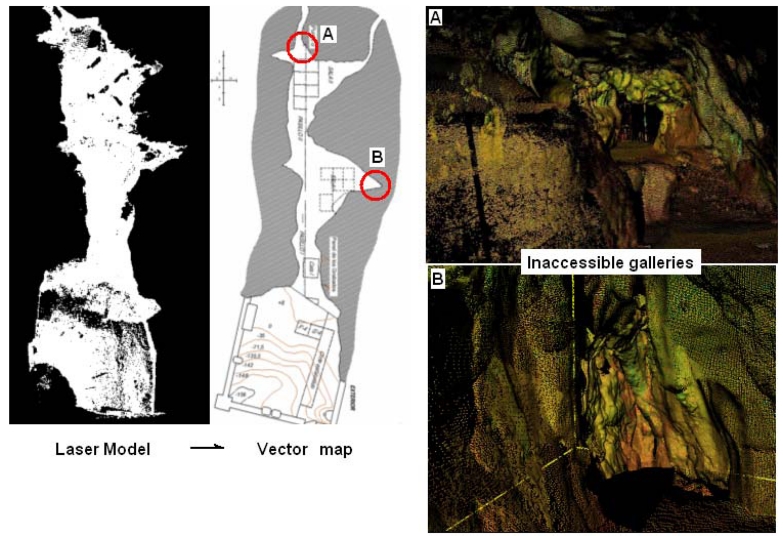
Left: Top view of the laser model at “Las Caldas” cave. Centre: Vector floor plan extracted from laser scanner dataset. Right: Inaccessible galleries recorded with TLS.

Up to now, it had been impossible to provide a complete map at “Las Caldas” cave due to the classical approaches based on manual measurements and instruments used. Thanks to TLS technology, several inaccessible galleries have been recorded and thus a general floor plan extracted from the laser scanner has been generated incorporating the archaeological grid and several reference measurements (Figure 4).

**Figure 5.**
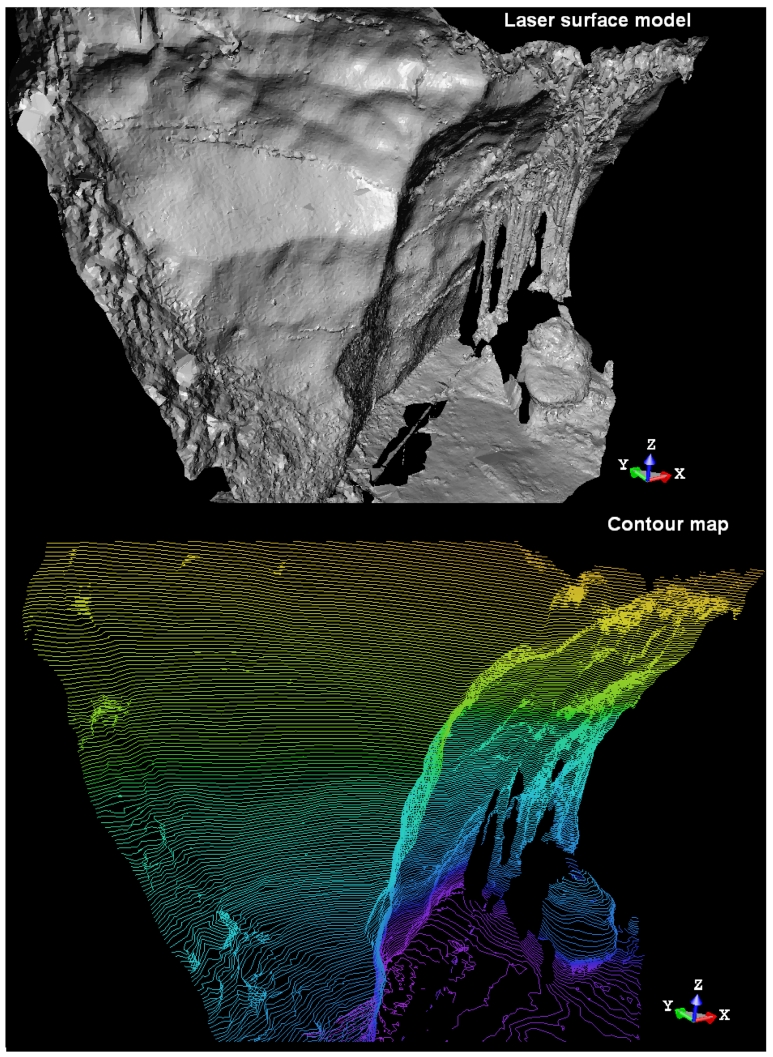
Laser surface model obtained from artificial shading (top) and contour map (down) extracted from this model at “Peña de Candamo” cave.

One of the most interesting possibilities available through the TLS is the amount of geometric information acquired, impossible to achieve by the classical surveying or cartographic techniques. In this way, several detailed surface models and contour maps that show the shapes and profiles of the caves with high accuracy are obtained automatically (Figure 5).

## 4. Discussion and Conclusions

In this paper, a multi-sensor approach for the 3D data acquisition and reconstruction of cave passages and chambers geometry has been presented. The characterization and description of caves, with great resolution and quality, opens a new way in underground surveying necessary to understand the speleogenesis processes and will become a widespread tool in whatever study to be performed in the underground world. Besides this, the final results obtained have been well accepted by pre-historians and archaeologists, since they usually obtain their metric measurements based only on classical approaches which are not very accurate and are very time consuming. Furthermore, the presented tool may be of crucial importance in preserving the cultural heritage from touristic overexploitation or other unknown hazards that might eventually deteriorate definitely the extremely fragile Paleolithic art forms.

On the other hand, authors have investigated and developed the automatic co-registration of two sensors: terrestrial laser scanner and conventional digital camera. Through this approach, a central issue for the integration of sensor technology has been solved efficiently using precise and reliable data processing schemes. With relation to the most relevant aspects of the proposed approach, we could remark on:

- A hierarchical and robust matching process that combines several proven scientific techniques in close range photogrammetry and computer vision has been developed and applied to real and complex rock art cases successfully.
- Particularly, the presented flowline exhibits the following improvements in comparison with other existing approaches discussed previously:
  - No user interaction is required.
  - No restrictions such as stereoscopic setup or physical attachment between both sensors are required.
  - Neither special targets nor geometric constraints (vanishing points) are necessary.

As a consequence, there is no doubt about the potential of this multi-sensor approach. However, several challenges remain and are the subject of future work:

- Use of new sensors (thermal and hyperspectral cameras) and filters for the digital camera in order to apply spectral analysis to the acquired data, could provide interesting results focused on pictorial materials, and the analysis of anomalous areas.
- Improving the feature extraction process developed. Particularly, the Scale Invariant Feature Transform (SIFT) approach developed by [32] could transform the camera and range images into a large collection of local feature vectors, each of which would be invariant to image translation, scaling, and rotation, and partially invariant to illumination changes and affine or 3D projection. This would have the further advantage that any change in perspective or rotation between both images would not affect the feature extraction process. Likewise, the procedure for co-registration of both sensors could be further improved by adding an approach for managing the occlusions problem.
